# Supplementary material for: Effect of Point Defects on Electronic Structure of Monolayer GeS
Source: Nanomaterials (Basel). 2021 Nov 4;11(11):2960. doi: 10.3390/nano11112960 (PMC8618743; doi:10.3390/nano11112960)
Supplement: Supplementary file 1 [file nanomaterials-11-02960-s001.zip › nanomaterials-1423962-supplementary.pdf]

nanomaterials

Supplementary Materials

Effect of Point Defects on Electronic Structure of Monolayer GeS

Hyeong-Kyu Choi, Janghwan Cha, Chang-Gyu Choi, Junghwan Kim and Suklyun Hong *

Department of Physics, Graphene Research Institute, and GRI-TPC International Research Center, Sejong University, Seoul 05006, Korea; chk75541@gmail.com (H.-K.C.); heatpoint@nate.com (J.C.); power_ccq@naver.com (C.-G.C.); uprightshine0415@naver.com (J.K.)

* Correspondence: Correspondence: hong@sejong.ac.kr

2 of 3

1. Atomic structures of pristine and defective GeS

Figure S1 shows perspective views of atomic structures of pristine and defective GeS for a better view. Some specific values of bond lengths are provided for comparison. Germanium and sulfur atoms are represented by green and yellow balls, respectively, while M (=S, Si, Sn) and X (=Se, Te) atoms are represented by blue and red ones, respectively.

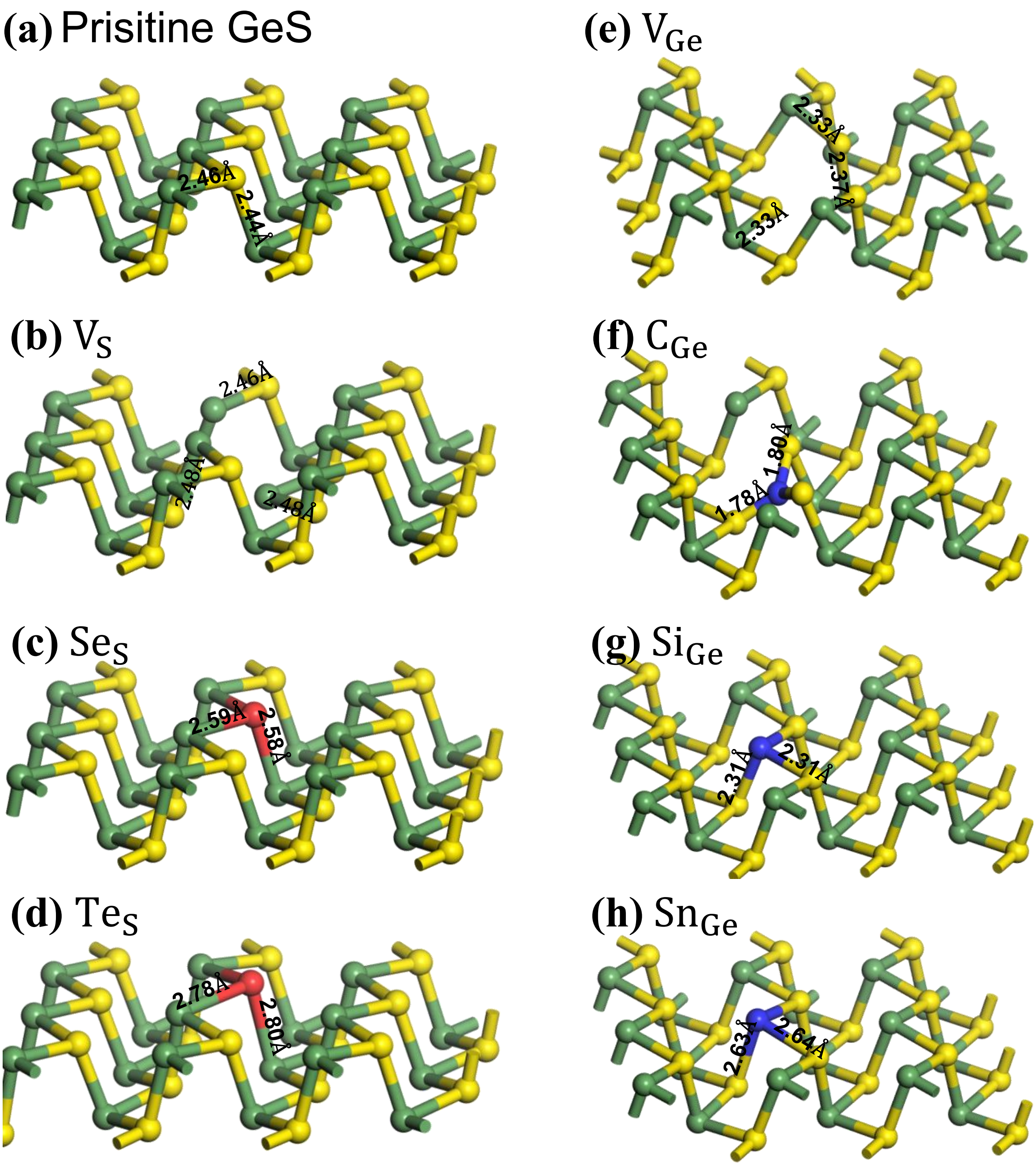

Figure S1. Perspective views of atomic structures of pristine and defective GeS.

3 of 3

2. Band structures of pristine and defective GeS

In Figure S2, band structures of defective GeS are provided along with that of pristine GeS for comparison. The band plots of defective GeS except V_{Ge}, V_S and C_{Ge} are very close to that of the pristine case: VBMs and CBMs of these structures are well defined and denoted by down and up arrows, respectively. For C_{Ge} and V_S, it is manifest that a localized state just below the Fermi level is closely related to defects, as shown in both band plots of Figure S2 and PDOS profiles of Figure 4. In addition, the band structures of V_{Ge} are much changed around the Fermi level, compared to the pristine case. Note that all the defective cases have indirect band gaps.

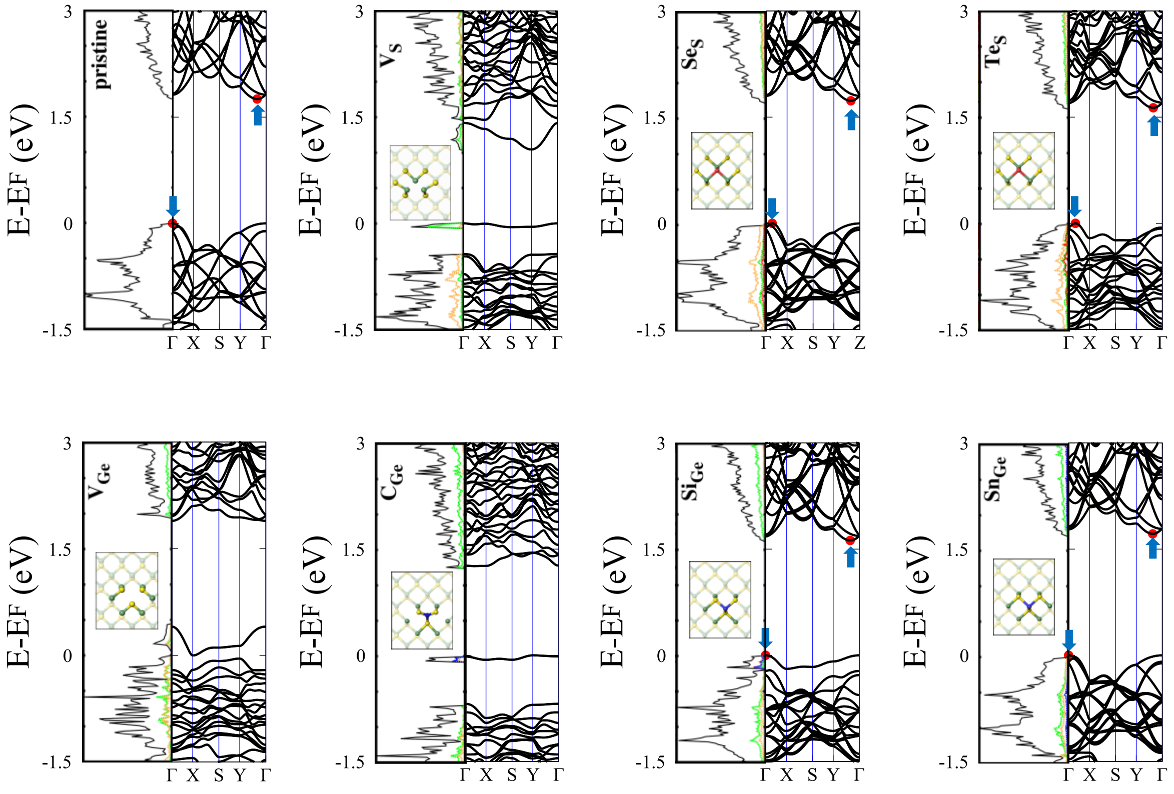

Figure S2. Band structures of pristine and defective GeS.
